# The influence of bone graft procedures on primary stability and bone change of implants placed in fresh extraction sockets

**DOI:** 10.1186/s40902-018-0148-2

**Published:** 2018-04-25

**Authors:** Sang Ho Jun, Chang-Joo Park, Suk-Hyun Hwang, Youn Ki Lee, Cong Zhou, Hyon-Seok Jang, Jae-Jun Ryu

**Affiliations:** 1 Department of Dentistry, Korea University Anam Hospital, Seoul, Republic of Korea; 2 Division of Oral and Maxillofacial Surgery, Department of Dentistry, College of Medicine, Hanyang University, Seoul, Republic of Korea; 3 Department of Medicine, Korea University Graduate School, Seoul, Republic of Korea; 4 Department of Dentistry, Korea University Ansan Hospital, Ansan-si, Republic of Korea; 5 Department of Advanced Prosthodontics, Korea University Anam Hospital, Inchon-ro 73, Seongbuk-gu, Seoul, 02841 Republic of Korea

**Keywords:** Immediate implant, Bone graft, Primary stability, Peri-implant bone change

## Abstract

**Background:**

This study was to evaluate the effect of bone graft procedure on the primary stability of implants installed in fresh sockets and assess the vertical alteration of peri-implant bone radiographically.

**Methods:**

Twenty-three implants were inserted in 18 patients immediately after tooth extraction. The horizontal gap between the implant and bony walls of the extraction socket was grafted with xenografts. The implant stability before and after graft procedure was measured by Osstell Mentor as implant stability quotient before bone graft (ISQ bbg) and implant stability quotient after bone graft (ISQ abg). Peri-apical radiographs were taken to measure peri-implant bone change immediately after implant surgery and 12 months after implant placement. Data were analyzed by independent *t* test; the relationships between stability parameters (insertion torque value (ITV), ISQ abg, and ISQ bbg) and peri-implant bone changes were analyzed according to Pearson correlation coefficients.

**Results:**

The increase of ISQ in low primary stability group (LPSG) was 6.87 ± 3.62, which was significantly higher than the increase in high primary stability group (HPSG). A significant correlation between ITV and ISQ bbg (*R* = 0.606, *P* = 0.002) was found; however, age and peri-implant bone change were not found significantly related to implant stability parameters. It was presented that there were no significant peri-implant bone changes at 1 year after bone graft surgery.

**Conclusions:**

Bone graft procedure is beneficial for increasing the primary stability of immediately placed implants, especially when the ISQ of implants is below 65 and that bone grafts have some effects on peri-implant bone maintenance.

## Background

Numerous studies over the past 20 years have confirmed the predictability and success of implant placement at the time of extraction [1–3], with a number of prospective and retrospective studies confirming high survival rates for more than 95% of immediate post-extraction implants and with observation periods ranging from 1 to 5 years [1, 4–7]. Albrektsson et al. [8] reported that primary implant stability and lack of micromovement are two of the main factors considered necessary for the achievement of predictably high success rates for osseointegrated oral implants. Primary stability of implants placed immediately after extraction strongly influences the long-term success of dental implants [9].

Following tooth extraction, the alveolar bone supporting tooth undergoes constant atrophy [10]. It is shown that a marked reduction of the height of the alveolar ridge consistently occurred following tooth extraction and that implant installation in the fresh extraction socket did not interfere with the process of bone modeling [11]. For an improved esthetic and functional prosthodontic result, it is necessary to preserve the alveolar bone volume after tooth extraction to facilitate the subsequent placement of dental implants [10]. Chen and Buser [12] reviewed 91 studies and concluded that bone augmentation procedures are effective in promoting bone fill and defect resolution for implants in post-extraction sites.

Although implant primary stability and spontaneous bone graft after immediate implantation are important factors for high success rates and esthetic outcomes, the relationship between them has not been sufficiently evaluated. The aim of the present study was to evaluate the effect of bone graft after immediate implant placement in fresh extraction socket.

## Methods

### Study sample

This study received approval from the ethics committee of the Korea University Anam Hospital, Seoul, South Korea (AN13025-002). We included a total of 23 implant sites in 18 patients who required immediate implant installation following tooth extraction in this study. All patients were healthy and had no uncontrolled systemic diseases.

After thorough diagnosis and treatment planning, immediate implant placement was performed under local anesthesia. A total of 23 implants (Straumann^®^ Bone Level SLActive^®^ Implants) with favorable initial stability were used in this study.

### Implant stability measurement

Implant placement was performed according to the sequences recommended by manufacturers (Straumann AG, Basel, Switzerland). Final seating torque value was measured as insertion torque value (ITV) (INTRAsurg 300, KaVo, Germany) during implant placement. In all cases, an intentional bone augmentation procedure was simultaneously performed with xenografts (Bio-Oss^®^, Geistlich, Switzerland) of 1–2-mm particle size into the gap between the implant fixture and the residual alveolar bone along the buccal and lingual surface. To standardize the bone graft procedure, a xenograft was packed into the gap by the controlled force minimizing the pulverization of the graft particles up to the remaining crestal level of the extraction socket. Resonance frequency analysis (RFA), using Osstell Mentor (Osstell AB, Gothenburg, Sweden), was performed to evaluate the primary stability of implants before and after bone grafting. Each implant was measured three times. The mean value of measurement before bone grafting was designated as the implant stability quotient before bone graft (ISQ bbg), and the mean measurement after bone graft was designated as the implant stability quotient after bone graft (ISQ abg). A healing abutment similar in diameter to that of the natural tooth was connected. The reflected flap was carefully sutured with 5-0 nylon.

### Peri-implant bone change analysis

**Fig. 1:**
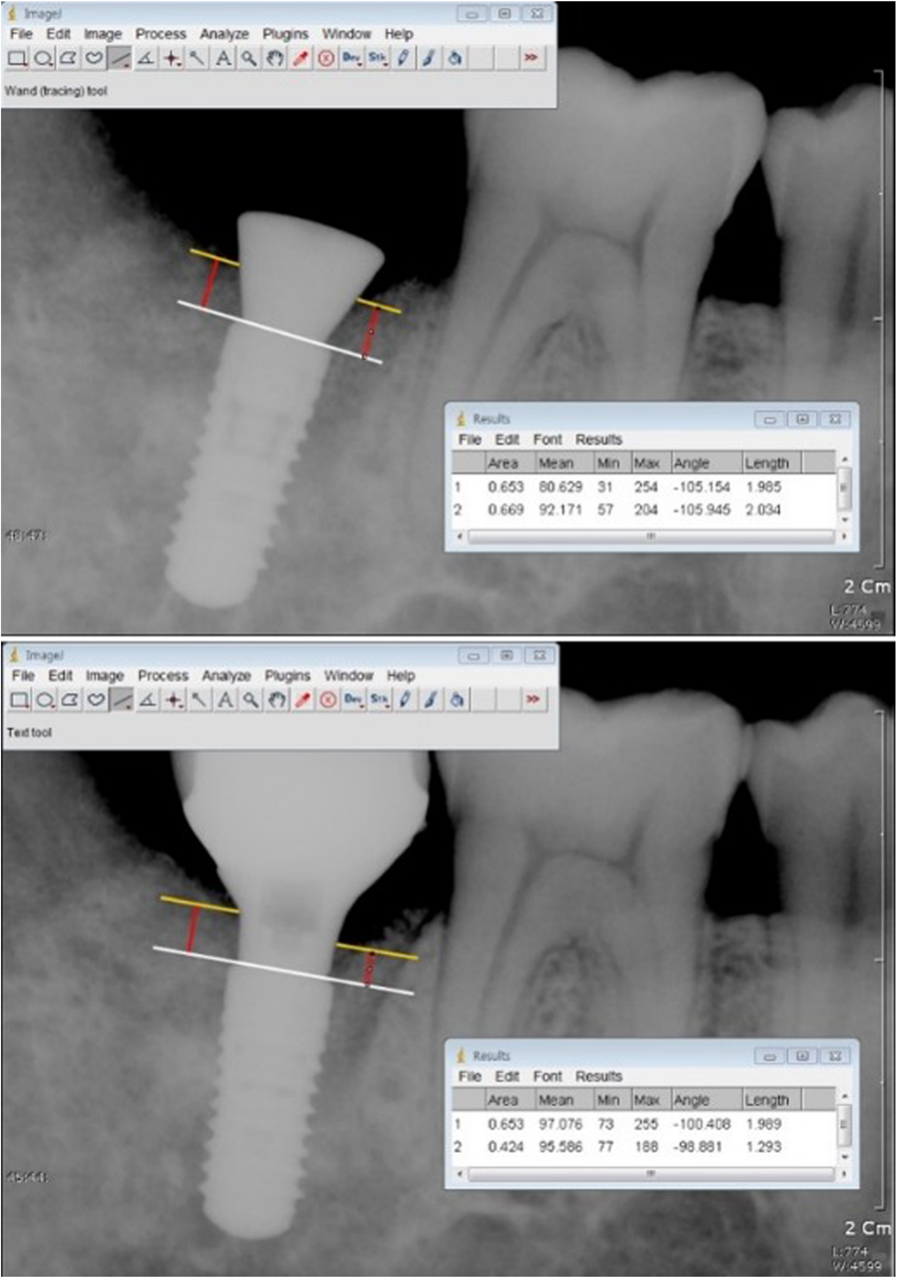
Peri-implant bone measurements, both after implant placement and at 1-year follow-up

To measure the peri-implant bone changes, peri-apical radiographs were taken both at the time of implant placement and 12 months after placement. Vertical measurements were taken from the mesial and the distal shoulder of the implant to the first bone-implant contact in an axis parallel to the implant (Fig. 1). A positive numerical value was recorded when the first bone-implant contact was higher than the implant shoulder, and a negative numerical value was recorded if the contact was lower than the implant shoulder. The data collected at the time of implant placement were used as the baseline value. Analysis of the peri-implant bone change was computed using image analysis software (ImageJ, 1.33i, National Institutes of Health, Bethesda, MD).

### Statistical analysis

Independent *t* test was used for the comparison of the mean values of ISQ abg and ISQ bbg and peri-implant bone changes according to the implantation sites, and the number of tooth roots was compared by one-way analysis of variance (ANOVA).

The relationships between the initial stability parameters (ITV, ISQ abg, and ISQ bbg) and peri-implant bone changes were analyzed according to Pearson correlation coefficients. All statistical analyses were performed using SPSS 19.0 statistical software (IBM SPSS, Armonk, NY). *P* values less than 0.05 were considered statistically significant.

## Results

The average age of the 18 patients was 46.1 years, with 11 male and 7 female patients. A total of 23 implants were installed in tooth extraction sockets and healed without complications. Follow-up appointments were scheduled until 12 months after implant placement in order to measure the peri-implant bone changes. Two cases did not meet follow-up visit criteria, and thus, the two implants in these two patients were excluded from measurement of peri-implant bone change. Patient information and the data of implant stability are listed in Table 1.

**Table 1:** Patient information and measurements of implant stability and bone changes after 1 year follow-up

No.	Age	Sex	Site	ITV (Ncm)	Mean ISQ		Bone changes (mm)
					bbg	abg	
1	50	M	#17	11	63	67.67	− 0.12
2	41	M	#11	18	75	75.33	− 0.58
3	59	F	#16	6.1	50.33	63.67	1.98
4	33	M	#15	5.4	41	53	Excluded
5	68	F	#16	17	61	66	0
6	68	F	#17	10	48	60.33	0
7	32	M	#16	13	53	60	1.82
8	39	M	#35	27	71.67	74.67	0
9	76	F	#36	10	59.33	66.67	0
10	46	M	#47	7.6	51.33	54.33	0
11	68	F	#44	20	71	74	1.21
12	68	F	#47	5.7	46	51	1.23
13	68	F	#22	15	75	76.67	1.78
14	28	M	#11	19	70	76.67	1.12
15	28	M	#21	8.2	69.67	69.67	1.43
16	15	F	#21	6.7	62.33	66.67	− 1.23
17	52	M	#26	14	76	79.67	− 1.34
18	28	M	#37	3.2	41	44	− 1.12
19	37	M	#22	8.9	74.33	75.33	Excluded
20	30	M	#37	14	42	50.33	− 1.55
21	30	M	#15	11	72.33	76	− 0.32
22	53	F	#36	16	62	66	− 0.55
23	66	F	#36	20	75	75.67	− 0.84

*ITV* insertion torque value, *ISQ* implant stability quotient, *bbg* before bone graft, *abg* after bone graft

### Statistical correlations among variables

**Fig. 2:**
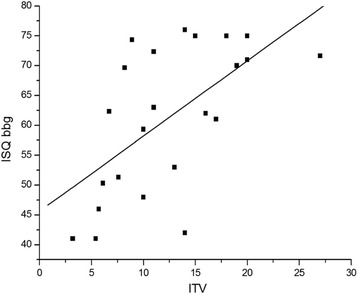
The correlation between implant stability quotient (ISQ) and insertion torque value (ITV)

There was a significant correlation between ITV and ISQ bbg (*R* = 0.606; *P* = 0.002) (Fig. 2). Patient age was not found to be significantly related to either implant stability or peri-implant bone change. There was also no significant correlation between peri-implant bone changes and implant stability parameters (ITV, ISQ bbg, and ISQ abg) (Table 2).

**Table 2:** Pearson’s correlations among patient age, implant stability parameters, and peri-implant bone changes

		Age	ITV	ISQ bbg	ISQ abg	Bone change
Age	*R*	1.000	0.187	0.069	0.147	0.254
	*P*		0.394	0.754	0.503	0.266
ITV	*R*	0.187	1.000	0.606	0.603	− 0.033
	*P*	0.394		0.002	0.002	0.886
ISQ bbg	*R*	0.069	0.606	1.000	0.964	0.036
	*P*	0.754	0.002		< .0001	0.879
ISQ abg	*R*	0.147	0.603	0.964	1.000	− 0.088
	*P*	0.503	0.002	< .0001		0.705
Bone level change	*R*	0.254	− 0.033	0.036	0.088	1.000
	*P*	0.266	0.886	0.879	0.705	

*ITV* insertion torque value, *ISQ* implant stability quotient, *bbg* before bone graft, *abg* after bone graft

**Fig. 3:**
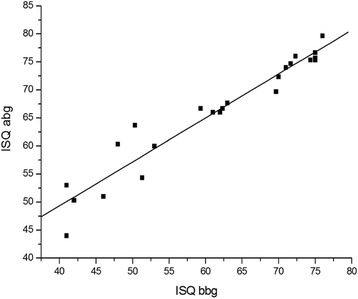
The correlation between implant stability quotient after bone graft (ISQ abg) and implant stability quotient before bone graft (ISQ bbg)

The average ISQ bbg was 61.32 ± 12.29 and ranged from 41.00 to 76.00; the average ISQ abg was 66.23 ± 10.16 and ranged from 44.00 to 79.67. A statistically significant increase of ISQ was observed 4.91 ± 3.75 (*P* < 0.001) away from the ISQ bbg. There was a strong correlation between the ISQ bbg and the ISQ abg (*R* = 0.96; *P* < 0.01) (Fig. 3).

### Influence of bone graft on implant primary stability

**Fig. 4:**
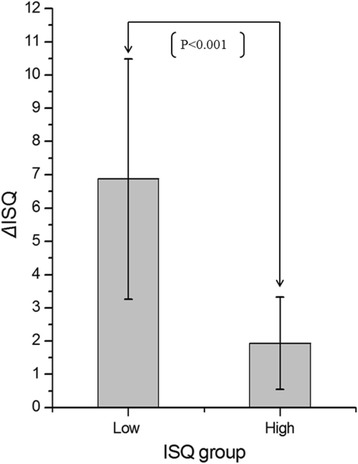
The mean increase of implant stability quotient (ISQ) after bone graft procedure in both the low and the high primary stability groups

**Fig. 5:**
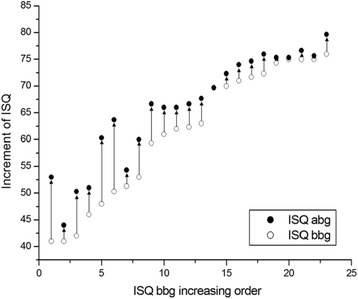
The changes of mean implant stability quotient (ISQ) for all the implants after bone graft procedures in both the low primary stability group (LPSG) and the high primary stability group (HPSG). The implants are arranged in the sequence of ISQ before bone graft (bbg). The mean ISQ bbg below 65 was defined as the LPSG, above 65 was defined as the HPSG

To investigate how bone graft procedure influenced the various implants’ primary stability, we defined those with a mean ISQ bbg below 65 as the “low primary stability group” (LPSG) and those with a mean ISQ bbg above 65 as the “high primary stability group” (HPSG). Thus, the ISQ bbg of HPSG ranged from 69.97 to 76.00 (mean value 73.00 ± 2.34), while the ISQ bbg of LPSG ranged from 41.00 to 63.00 (mean value 52.33 ± 8.46). The increase in implant stability after bone graft was significantly higher in the LPSG (6.87 ± 3.62) than in the HPSG (2.368 ± 2.05) (*P* = 0.002) (Figs. 4 and 5).

### Peri-implant bone changes

We compared the radiograph images from the day of implant installation with those taken at the 1-year follow-up. The mean change of peri-implant bone was 0.14 ± 1.11 mm in 1 year. The one-sample *t* test revealed no significant peri-implant bone changes at 1 year post-implantation (*t* = 0.57; *P* = 0.57). In addition, no statistically significant differences in peri-implant bone changes were found according to the implantation sites and the number of tooth roots.

## Discussion

Immediate installation of implants in fresh sockets is a challenging and sensitive technique and requires careful case selection [13]. It has been postulated that when the size of horizontal gap surrounding immediately placed implants exceeds the threshold of 1 to 2 mm, bone graft procedures might be recommended to reduce peri-implant bone resorption and improve the esthetic outcome of the soft tissue [10, 12, 14, 15]. The influence of grafting procedure on primary stability of immediately placed implants, nevertheless, has not been given enough attention.

Though RFA suffers from a lack of sensitivity to the quality of surrounding bone, ISQ measurement is a versatile technique that can be used repeatedly for quantitative stability both intraoperatively and postoperatively [16]. We found a strong correlation between ITV and ISQ in accordance with the previous study that analyzed the ITV and ISQ on a fresh cadaver and concluded that ITV and ISQ were statistically correlated [16]. In another experiment carried out in fresh-frozen pig femoral bones, Zhou et al. [17] obtained a similar result to the present study that correlation between ISQ and ITV was significant. Some studies, however, failed to demonstrate a statistical correlation between ISQ and ITV [18, 19], although the ITV was obtained using some equipment, such as implant engines and various custom-made manual torque devices, which may have affected their results. No significant correlation between peri-implant bone changes and implant stability parameters (ITV, ISQ bbg, and ISQ abg) was detected by the present study. In addition, implantation sites and defect configurations classified by the number of tooth roots showed no significant effects on peri-implant bone changes after the immediate placement of implant. However, a consensus has been reached that bone graft procedure is beneficial in preventing peri-implant bone loss, which was confirmed by this study. Araújo et al. [10] evaluated the osseointegration and peri-implant tissue modeling following implant placement in fresh extraction sockets, and he found that in the absence of bone graft, the dimensions of both the buccal and the lingual bone walls around the implant were reduced; even the osseointegration could be in part lost following bone modeling. Therefore, with the support from experimental evidence, the control group in which bone graft procedure would not be carried out was not included in this study.

In the present study, the xenografts filled the horizontal gap between the bony walls and the implants. It is worth noting that the grafting procedure led to an increase in the primary stability of immediately placed implants, which was more notable for implants in the LPSG, in which the ISQ bbg was below 65 (Fig. 4). In light of the effect of increasing the primary stability of implants, grafting the horizontal gap with bone substitutes could be beneficial. The influence of graft procedure on implant primary stability has not been intensively studied, even though Santos et al. [20] evaluated recent animal and human studies of bone substitutes used for peri-implant defects in post-extraction implants. They concluded that although the technique of installing implants in fresh extraction sockets is a reliable alternative to reduce treatment time, the use of a biomaterial is required to increase bone-implant contact and enhance osseointegration.

It has been proven that alveolar bone remodeling appeared progressively active following tooth extraction; the horizontal resorption of the buccal alveolar dimension amounted to about 56% at 4 months after tooth loss [21]. Based on indications from McGlumphy and Larsen [22], when the size of the gap is less than 1 mm, the graft procedure is not needed; it is only when the size of gap exceeds 1 mm that bone graft or other guided bone regeneration procedures may be necessary. Although several kinds of bone graft materials, such as autogenous graft, allograft, xenograft, synthetic materials, or any combination of these, have been used or tested to maintain the bone level around immediately placed implants, none have been proven to be superior to the others [20, 23, 24]. In this study, bovine bone was used as the grafting material to augment the horizontal gaps adjacent to immediate implants. We found that the bundle bone was progressively resorbed during the modeling and remodeling stages of the extraction healing process, similar to the previous finding that bundle bone could be observed only until 2 weeks after tooth extraction [25, 26]. It has been found that xenografts play an important part in alveolar bone preservation and can maintain the dimensions of the extraction socket instead of the bundle bone, as well as encourage osteoconduction and space maintenance [27]. In our study, the graft procedure resulted in no marginal bone loss after 1 year of follow-up, which was similar to findings in previous studies [15, 23].

## Conclusions

The results of this study indicate that the bone graft procedure is beneficial for increasing the primary stability of immediately placed implants, especially when the ISQ of implants is below 65. Both ITV and ISQ are effective and practical methods detecting implant primary stability and are statistically correlated with each other.

## Abbreviations

HPSG: High primary stability group
ISQ abg: ISQ after bone graft
ISQ bbg: ISQ before bone graft
ISQ: Implant stability quotient
ITV: Insertion torque value
LPSG: Low primary stability group
